# Plant-soil-enzyme C-N-P stoichiometry and microbial nutrient limitation responses to plant-soil feedbacks during community succession: A 3-year pot experiment in China

**DOI:** 10.3389/fpls.2022.1009886

**Published:** 2022-09-20

**Authors:** Hongwei Xu, Qing Qu, Zhanhui Wang, Sha Xue, Zhenfeng Xu

**Affiliations:** ^1^National Forestry and Grassland Administration Key Laboratory of Forest Resources Conservation and Ecological Safety on the Upper Reaches of the Yangtze River, Forestry Ecological Engineering in the Upper Reaches of the Yangtze River Key Laboratory of Sichuan Province, Sichuan Agricultural University, Chengdu, China; ^2^State Key Laboratory of Soil Erosion and Dryland Farming on Loess Plateau, Institute of Soil and Water Conservation, Northwest A&F University, Yangling, China; ^3^Hebei Drinking Water Safety Monitoring Technol Inn, Chengde, China

**Keywords:** microbial metabolic limitation, vector length, vector angle, species substitution, plant community, Loess Plateau

## Abstract

Studying plant-soil feedback (PSF) can improve the understanding of the plant community composition and structure; however, changes in plant-soil-enzyme stoichiometry in response to PSF are unclear. The present study aimed to analyze the changes in plant-soil-enzyme stoichiometry and microbial nutrient limitation to PSF, and identify the roles of nutrient limitation in PSF. *Setaria viridis*, *Stipa bungeana*, and *Bothriochloa ischaemum* were selected as representative grass species in early-, mid-, and late-succession; furthermore, three soil types were collected from grass species communities in early-, mid-, and late-succession to treat the three successional species. A 3-year (represents three growth periods) PSF experiment was performed with the three grasses in the soil in the three succession stages. We analyzed plant biomass and plant-soil-enzyme C-N-P stoichiometry for each plant growth period. The plant growth period mainly affected the plant C:N in the early- and late- species but showed a less pronounced effect on the soil C:N. During the three growth periods, the plants changed from N-limited to P-limited; the three successional species soils were mainly limited by N, whereas the microbes were limited by both C and N. The plant-soil-enzyme stoichiometry and plant biomass were not significantly correlated. In conclusion, during PSF, the plant growth period significantly influences the plant–soil–microbial nutrient limitations. Plant-soil-enzyme stoichiometry and microbial nutrient limitation cannot effectively explain PSF during succession on the Loess Plateau.

## Highlights

- Plants changed from nitrogen- to phosphorus-limited during the plant growth period.
- Soil C:P and N:P overall increased during the plant growth period.
- Soil microbes are limited by both carbon and nitrogen in the PSF process.
- The carbon limitation of microbes decreased during the plant growth period.
- PSF effect cannot be explained by the plant-soil-enzyme stoichiometry.

## Introduction

Plant-soil feedback (PSF) is defined as a relationship in which plant growth changes the soil biotic and abiotic properties, which in turn, induce positive, neutral, or negative feedback effects on the original plant species or other plant species (Bever et al., 1997; Klinerova and Dostal, 2020). Ecosystem structure and community succession have been examined extensively from the perspective of PSF (van de Voorde et al., 2011; Zhang et al., 2016; Wilschut and van Kleunen, 2021). During plant community succession, different successional stages species are subject to different PSF effects, which promote species replacement in the community (Kardol et al., 2006). Therefore, studying PSFs can advance the understanding of PSF effects during community succession.

Ecological stoichiometry is a novel aspect of ecosystem research which can provide insights into plant-soil interactions and chemical cycles (Elser et al., 1996; Sterner and Elser, 2002; Frazao et al., 2020; Xu et al., 2022). Although ecological stoichiometry studies revealed that organisms can maintain relatively stable carbon (C), nitrogen (N), and phosphorus (P) compositions (Sistla and Schimel, 2012), changes in environmental factors can also affect the elemental stoichiometry of organisms (Fang et al., 2019). For example, plant community succession involves changes in the community over time (Kristiina et al., 2014). Yang and Luo (2011) showed that the plant C:N increased significantly during succession, whereas the soil C:N maintained a higher steady state. The soil enzyme stoichiometry can also be used to characterize microbial nutrient requirements and limitations (Sterner and Elser, 2002). During succession, soil microorganisms are limited by C, P, or both of them C and P (Xu et al., 2019; Liu et al., 2020). Soil C, N, and P, as well as their stoichiometry, are significantly related to the nutrient limitation of plants and microorganisms (Fang et al., 2019; Klinerova and Dostal, 2020; Xu et al., 2021a).

Current research on PSF mainly focuses on changes in plant biomass, soil nutrients, and microbe diversity (Michaels, 2003; van de Voorde et al., 2011; Gundale et al., 2019). Some studies use changes in plant biomass to determine the direction and magnitude of PSF effects (Kardol et al., 2006; Huberty et al., 2020), and explain the mechanism of PSF changes from the perspective of changes in soil nutrients and microbial activity (Png et al., 2018; Wang et al., 2019). However, plant growth can affect soil nutrients through nutrient usage and litter return (Miki and Kondoh, 2002). Moreover, plant growth can markedly affect soil microbial community structures (Zhao et al., 2021), thus affecting the uptake of nutrients by plants and changing the geochemical characteristics of the ecosystem (van der Putten et al., 2013; Ren et al., 2015). For example, plant species exerting negative PSF effects show higher nutrient usage (Miki and Kondoh, 2002; Crawford et al., 2019). Mycorrhizal fungi play important roles in positive PSF processes as they can stimulate plants to consume soil nutrients (Orwin et al., 2011), and changes in nutrient content can affect the microbial nutrient limitation status (Johnson, 2010). However, it remains unclear how PSF mechanisms affect changes in plant-soil-enzyme C-N-P stoichiometry. Determining plant-soil-enzyme C-N-P stoichiometry and microbial nutrient limitation is important for understanding the equilibrium of chemical components in the ecosystem and nutrient regulation (Michaels, 2003; Xu et al., 2022).

In this study, three species representing different succession stages (i.e., early-, mid-, and late-succession species) were selected from a typical secondary ecological succession on the Loess Plateau, China. We conducted a PSF experiment for 3 years (representing three plant growth periods) to examine changes in plant-soil-enzyme C-N-P stoichiometry and microbial nutrient limitation due to PSF and to identify the roles of nutrient limitation in PSF. The key objectives were (1) to elucidate the changes in plant-soil-enzyme C-N-P stoichiometry and microbial nutrient limitation during the three plant growth periods; (2) to reveal the relationships underlying C-N-P stoichiometry of plants, soil, and soil enzymes in the three plant growth periods; and (3) to verify whether plant-soil-enzyme C-N-P stoichiometry and microbial nutrient limitation can explain the PSF effects of different species during succession. This study provides a theoretical basis for PSF during community succession from the perspective of plant-soil-microbe nutrient absorption and nutrient limitation.

## Materials and methods

### Experimental design

*Setaria viridis* (Sv), *Stipa bungeana* (Sb), and *Bothriochloa ischaemum* (Bi) have been selected as study species to study the C-N-P stoichiometry of plant-soil-enzyme and the microbial nutrient limitation response to PSF during succession. Sv, Sb, and Bi are representative dominant early-, mid-, and late- successional species, respectively, on the Loess Plateau (Zhang et al., 2021).

The Ansai Research Station of the Chinese Academy of Sciences (36°51′ N, 109°19′ E, 1,068–1,309 m a.s.l.) belongs to the long-term location detection sampling sites, and thus, there are grassland communities with single species composition of different succession stages. The mean annual precipitation and temperature in this region are 505.3 mm and 8.8^°^C, respectively. The soil type is Inceptisol (according to the USDA Soil Taxonomy) or Cambisol (according to World reference base system).

Five 20 m × 20 m plots of the early- species (farmland fields abandoned for 1–2 years to form grasslands), five 20 m × 20 m plots of the mid- species (farmland fields abandoned for 10–20 years), and five 20 m × 20 m plots of the late- species (farmland fields abandoned for > 30 years) were selected to collect seeds and soil (see Supplementary Table 1 for more detail). A total of 15 plots was selected. Seeds of the three species were obtained from adult plants. Before the PSF experiment, the seeds were sterilized with sodium hypochlorite (2%) for 1 min, rinsed 5 times with deionized water, and placed in an alcohol (70%)-sterilized petri dish for germination in an incubator.

From April 25 to 30, 2018, soil samples were obtained (early, mid, and late soil, representing the three succession stages). Briefly, the ground litter and top 0–5 cm soil layer were removed, and then the root-zone soil (5–15 cm deep) was collected and used as the experimental soil. Briefly, the whole plant root mass was dug out with a shovel and gently shaken, and soil loosely and closely associated with the rhizosphere was collected. The collected soils were sieved (0.5 cm) in the laboratory, and the root-zone soils of the 5 plots of the same species were fully mixed by manual stirring for pot planting and stored at 4^°^C until use (Jing et al., 2015; Wang et al., 2019). The average chemical and biological properties of field soils that support early-, mid-, and late- species are shown in Supplementary Table 2. The PSF experiment was performed at the State Key Laboratory of Soil Erosion and Dryland Farming on the Loess Plateau, Institute of Soil and Water Conservation, Chinese Academy of Sciences and Ministry of Water Resources (34°12′ N, 108°07′ E, 530 m a.s.l.) in a greenhouse.

### Plant-soil feedback experiment

To study the plant-soil-enzyme C-N-P stoichiometry and microbial nutrient limitation response to PSF, Sv, Sb, and Bi were planted on May 4, 2018 into early, mid, and late soil, comprising 9 treatments, with 18 pots per treatment (18 replicates) and a total of 162 pots (Supplementary Figure 1A).

First, 1 kg of crushed stones (diameter approximately 0.1 cm) was placed at the bottom of a pot (inner diameter × height, 20 × 15 cm) to prevent soil compaction (Zhang et al., 2021). A plastic tube with a diameter of 1 cm and length of 25 cm was vertically inserted as a watering channel, and a layer of paper was placed on top of the stones to separate the soil and crushed stone (Supplementary Figure 1B).

To examine the growth of the three species in the three soils, Sv, Sb, and Bi were grown separately in the early, mid, and late soil (2.8 kg/pot). Approximately 10 seeds of each species were sown into each pot along with sufficient water to promote seed germination. At 2 weeks after sowing, thinning was performed, leaving four plants per pot (based on vegetation density from field surveys).

During the growth period, the pot positions were randomly exchanged once per week, and the water content was controlled twice per week (80% of the field water-holding capacity). After 4 months of growth, five pots were randomly selected from each treatment for the plant and soil samples harvesting. To simulate temporal PSF during the continuous growth of plants under conditions similar to those in the field site, the aboveground parts of the perennial plants (Sb and Bi) in the other pots were cut, and the underground part of the plant was retained. These pots were placed under the same environmental conditions as in the first growth period to prepare for the second year of the experiment. In the second year, Sb and Bi were not replanted but regrown from the previous year’s root. Because Sv is an annual plant, we replanted this species on May 4 of each year while retaining the root. After 4 months of plant growth, five pots from each treatment were randomly selected for sampling. This procedure was followed in the third year (see Supplementary Table 3 for more detail).

### Plant and soil sample collection

Plant and soil samples of the randomly selected pots (9 treatments × 5 pots = 45 pots/year) were collected on September 4 every year starting in 2018. Briefly, dust was removed from the plant leaves, the shoot was cut off at the soil surface with scissors, and the root was dug out. The soil attached to the root was washed with distilled water, and both the shoot and root were placed in a paper bag and dried at 105^°^C for 30 min and then at 70^°^C to a constant weight. The aboveground parts were pulverized with a grinder and sieved (1 mm) to determine the plant C, N, and P contents. After removing the underground part of the plant, the remaining soil in the pot was thoroughly mixed to homogeneity, passed through a 2 mm sieve, and divided into two parts. One part was air-dried naturally for analysis the soil chemical properties, and the second part was stored at 4^°^C until determination of the soil microbial biomass and enzyme activity.

### Chemical and biological analyses

The C, N, and P contents of the plants and soils were determined using the Walkley and Black (Nelson and Sommers, 1982), Kjeldahl (Bremner and Mulvaney, 1982), and molybdenum blue methods (Schade et al., 2003), respectively. Soil available nitrogen (AN) was determined using the Kjeldahl method (Bremner and Mulvaney, 1982), and soil available phosphorus was extracted with 0.5 M NaHCO_3_ (Olsen and Sommers, 1982). Soil microbial biomass, measured as the microbial biomass carbon (MBC) and microbial biomass nitrogen (MBN), was determined using the chloroform fumigation-extraction method (Vance et al., 1987), in which two soil samples were fumigated with chloroform for 24 h and two were not fumigated. An automatic organic carbon analyzer was used to determine the MBC, and an ultraviolet spectrophotometer was used to determine the MBN. The activity of the C-acquiring enzymes β-1,4-glucosidase (BG) and β-D-cellobiosidase (CBH), N-acquiring enzymes β-1,4-N-acetylglucosaminidase (NAG) and L-leucine aminopeptidase (LAP), and P-acquiring enzyme acid phosphatase (AP) were determined as described by Saiya-Cork et al. (2002) and Zhang et al. (2019). Briefly, 1.0 g of soil sample was weighed before adding 125 ml of 50 mmol/L CH_3_COOH buffer of the same pH as the bathing soil sample. After shaking, the soil solution was incubated at room temperature, and fluorescence was measured using a multifunctional microplate reader.

### Data analyses

The PSF index was selected to characterize the direction and strength of feedbacks in different species with three growth periods. The PSF index was calculated as follows (Baxendale et al., 2014):


$$\mathrm{PSF}\,\mathrm{index}=\frac{\left({\text{biomass}}_{\text{t}+1}-{\text{biomass}}_{\text{t}}\right)}{{\text{biomass}}_{\text{t}}}$$

where biomass_t_ is the biomass of the previous plant growth period, and biomass_t+1_ is the biomass of the next plant growth period, t = 1 or 2.

The plant and soil C-N-P stoichiometry ratios were based on their content ratio. The soil enzyme C-N-P stoichiometry is the ratio of C-, N-, and P-acquiring enzymes. To quantify the limitations of soil microbial metabolism of C, N, and P, vector analysis was performed to evaluate the relative proportion of ecological enzyme activities. Moorhead et al. (2013) described that the vector length and angle calculated by enzyme stoichiometry can reflect the relative P and N limitations of soil microbes. The vector length and angle were calculated as follows (Moorhead et al., 2013, 2016):


(1) $$\mathrm{Vector}\mathrm{length}={\text{SQRY[x}}^{2}\times {\text{y}}^{2}]$$

(2) Vectorangle()°=Degrees(Atan2[x,y])

Where *x* represents the relative activities of C- vs. P-acquiring enzymes and was calculated as (BG+CBH)/(BG+CBH+NAG+LAP); and *y* represents the relative activities of C- vs. N-acquiring enzymes and was calculated as (BG +CBH)/(BG+CBH+ AP).

One-way ANOVAs was used to evaluate the response of the plant-soil-enzyme C-N-P contents, plant-soil-enzyme C-N-P stoichiometry, and microbial nutrient limitation to plant growth period (*p* < 0.05). *T-*tests were conducted to evaluate the responses of the PSF index to different growth periods (*p* < 0.05). Before analysis, all data were tested for normality with Kolmogorov-Smirnov procedures (Lilliefors) and homogeneity with Levene’s test. Data that were not normally distributed were subjected to log (X+1) transformation. The effects of soil and microbial characteristics on plant-soil-enzyme C-N-P stoichiometry were tested using redundancy analysis (RDA) in Canoco software 5.0 (permutations = 999). Pearson correlation analysis was performed to investigate the relationships between plant shoot biomass and plant-soil-enzyme C-N-P stoichiometry. Figures were drawn using Origin v. 9.0 (OriginLab Corp., Northampton, MA, United States).

## Results

### Plant-soil feedback index

The PSF of the early- species were negative in the three soils, and the PSF index of first-second was higher than that of second-third in early and late soil (Supplementary Figure 2A). The PSF of the mid- species was natural but that of the later- species changed from positive to negative (Supplementary Figures 2B,C).

### Plant and soil C-N-P stoichiometry

**FIGURE 1:**
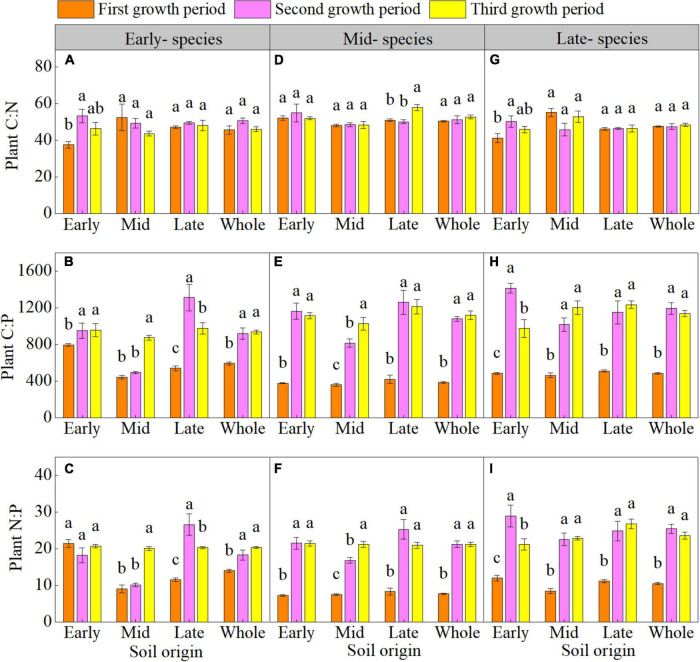
Mean (± SE) (*n* = 5) plant carbon-nitrogen-phosphorus stoichiometry (plant C:N, plant C:P, and plant N:P) of early- **(A–C)**, mid- **(D–F)**, and late- species **(G–I)** over the three growth periods. “Whole” is the average plant C:N, plant C:P, and plant N:P of the same species in the three soils. Different lower letters indicate significant differences in mean plant C:N, plant C:P, and plant N:P among the three growth periods based on Duncan’s *post-hoc* test (*p* < 0.05).

The plant C:N of early- and late- species in early soil significantly increased during the second growth period (Supplementary Table 4 and Figures 1A,G). The plant C:P of early- species in early and late soil; plant N:P of early- species in late soil; and plant C:P and N:P of mid- and late- species in early, mid, and late soil were higher overall in the second and third growth period than in the first growth period (Supplementary Table 4 and Figures 1A–I).

**FIGURE 2:**
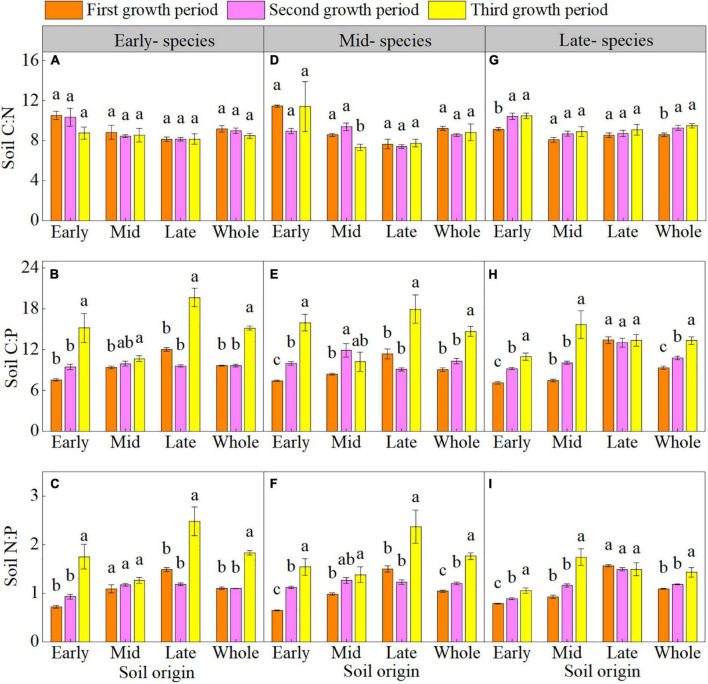
Mean (± SE) (*n* = 5) soil carbon-nitrogen-phosphorus stoichiometry (soil C:N, soil C:P, and soil N:P) of early- **(A–C)**, mid- **(D–F)**, and late- species **(G–I)** over the three growth periods. “Whole” is the average soil C:N, soil C:P, and soil N:P of the same species in the three soils. Different lower letters indicate significant differences in mean soil C:N, soil C:P, and soil N:P among the three growth periods based on Duncan’s *post-hoc* test (*p* < 0.05).

The plant growth period had minimal effects on the soil C:N but significant effects on the soil C:P and N:P in early- and mid- species. The soil C:P and N:P of early- and mid- species in early and late soil were higher in third growth period than in the first and second growth periods (Supplementary Table 4 and Figures 2A–F). The plant growth period affected the soil C:P and N:P of only late- species in early and mid-soil, which were higher overall in the third growth period (Supplementary Table 4 and Figures 2G–I).

### Enzyme C-N-P stoichiometry

**FIGURE 3:**
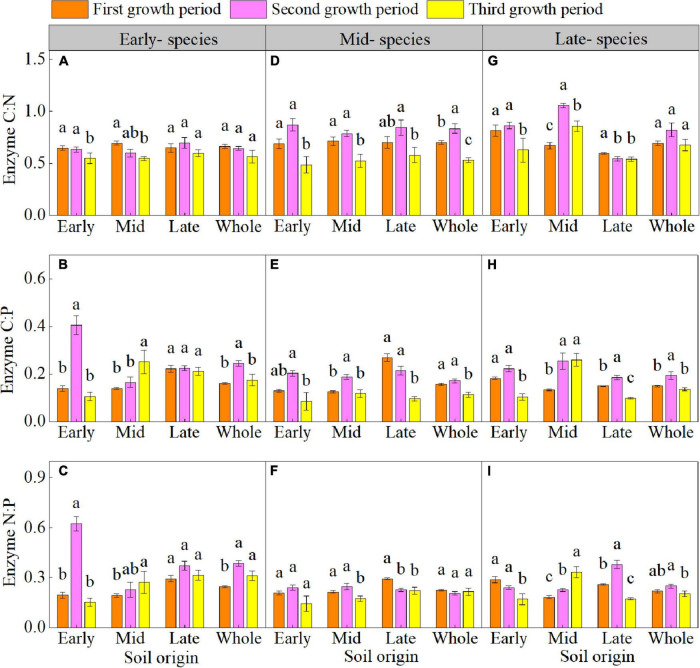
Mean (± SE) (*n* = 5) enzyme carbon-nitrogen-phosphorus stoichiometry (enzyme C:N, enzyme C:P, and enzyme N:P) of early- **(A–C)**, mid- **(D–F)**, and late- species **(G–I)** over the three growth periods. “Whole” is the average enzyme C:N, enzyme C:P, and enzyme N:P of the same species in the three soils. Different lower letters indicate significant differences in mean enzyme C:N, enzyme C:P, and enzyme N:P among the three growth periods based on Duncan’s *post-hoc* test (*p* < 0.05).

**FIGURE 4:**
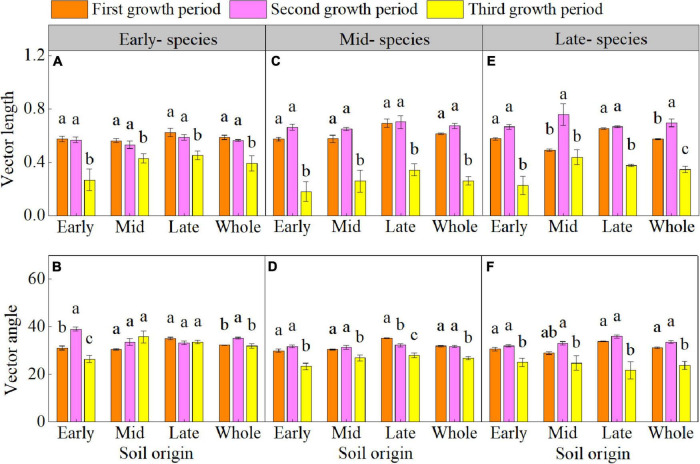
Mean (± SE) (*n* = 5) vector length and vector angle of early- **(A,B)**, mid- **(C,D)**, and late-species **(E,F)** over the three growth periods. “Whole” is the average vector length and vector angle of the same species in the three soils. Different lower letters indicate significant differences in mean vector length and vector angle among the three growth periods based on Duncan’s *post-hoc* test (*p* < 0.05).

The plant growth period significant affected the enzyme C-N-P stoichiometry (Supplementary Table 4 and Figures 3A–I). The enzyme C:N of early- species in early soil; enzyme C:N and C:P of mid- and late- species in early, mid, and late soils (except for the enzyme C:N of late- species in late soil); enzyme N:P of early- species in early soil; and enzyme N:P of late- species in early and late soil decreased in the third growth period. The vector length of three species and vector angle of mid- and late- species in early, mid, and late soil were lower in the third growth period than in the first and second growth periods (Figures 4A–F). The vector length and vector angle showed a significant positive correlation (Supplementary Figure 3).

### Factors affecting plant-soil-enzyme C-N-P stoichiometry

**FIGURE 5:**
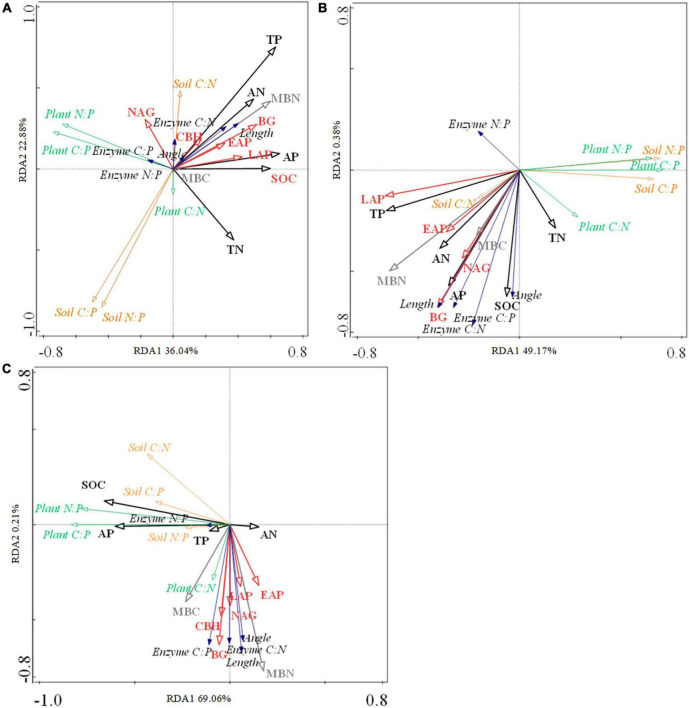
Relationships between soil nutrients (black), soil microbial biomass (gray), and soil enzyme activity (red) and plant (green)—soil (pink) -enzyme (blue) C-N-P stoichiometry in early- **(A)**, mid- **(B)**, and late-species **(C)**. SOC, soil organic carbon. TN, soil total nitrogen. TP, soil total phosphorus. AN, soil available nitrogen. AP, soil available phosphorus. MBN, soil microbial biomass nitrogen. MBC, soil microbial biomass carbon. BG, β-1,4-glucosidase. NAG, β-1,4-N-Acetylglucosaminidase. CBH, β-D-Cellobiosidase. MAP, Acid phosphatase. LAP, L-Leucine aminopeptidase.

RDA showed that soil chemical (SOC, TN, TP, AN, and AP) and microbial factors (MBC, MBN, BG, NAG, CBH, MAP, and LAP) explained 52.3, 49.6, and 69.3% of the total variation in early-, mid- and late- species, respectively (Figures 5A–C and Supplementary Table 5). Figures 5A,B (early- and mid- species) shows that the plant C:P, plant N:P, soil C:P, and soil N:P were negatively related to soil nutrient and enzyme activity. Figure 5B (mid- species) shows that the plant C:N, soil C:N, soil C:P, enzyme C:N, enzyme C:P, enzyme N:P, vector length, and vector angle were overall positively related to soil nutrient and enzyme activity. Figure 5C (late- species) shows that the soil C:P and N:P were negatively related to soil enzyme activity.

Except for the relationship of early- species biomass with plant N:P, the mid- species biomass with plant C:N and soil C:N and late- species biomass with plant C:P, plant N:P and vector length, the plant biomass and plant-soil-enzyme C-N-P stoichiometry were not significantly correlated (Table 1).

**TABLE 1:** Relationships of shoot biomass with plant, soil, and enzyme C-N-P stoichiometry of early-, mid-, and late- species (*n* = 45).

Species	Plant C:N	Plant C:P	Plant N:P	Soil C:N	Soil C:P	Soil N:P	Enzyme C:N	Enzyme C:P	Enzyme N:P	Vector length	Vector angle
Early-	0.02	0.08	0.33*	–0.20	–0.26	0.09	0.00	–0.11	–0.11	0.18	–0.06
Mid-	0.39**	0.00	–0.09	0.37*	0.15	–0.04	0.12	0.13	–0.03	0.09	0.23
Late-	–0.24	0.54**	0.60**	0.27	0.10	0.03	0.07	0.22	0.22	0.36*	0.11

Data are Pearson coefficient. Bold text indicates the relationships reach a significant level.

*p < 0.05, **p < 0.01.

## Discussion

### Plant C-N-P stoichiometry response to plant-soil feedback

Our study demonstrated that the plant C:N of early- and late- species in early soil significantly increased in the second growth period. Studies have confirmed that the nutrients absorption by plants is affected by soil nutrients availability (Li et al., 2021; Xu et al., 2021a). Early species showed a negative PSF (Figure 1A), mainly because soil nutrients were consumed and soil enzyme activity was reduced (Supplementary Figures 4, 5). Meanwhile, as early- species are annuals, the nutrient content in soil may decrease due to the removal of live roots of plants (Norby and Jackson, 2000; Jha and Mohapatra, 2010), which may be act as a negative PSF mechanism in early- species. The consumption of soil nutrients may reduce the nutrients absorption and utilization by plants. Moreover, in this study, the plant nitrogen content is at a relatively low level and lower than the average nitrogen level in China (18.6 g kg^–1^; Han et al., 2005), which further confirmed the above conclusion. For late- species, the PSF pattern changed from positive to negative during plant growth period (Figure 1C), possibly because soil nutrients accumulated in the second growth period and soil microbial activity was decreased in the third growth period (Supplementary Figure 6). Thus, the accumulation of soil nutrients may promote the accumulation of plant carbon (Supplementary Figure 4), whereas the plant growth period has little effect on the nitrogen content, resulting in an increased plant C:N. In addition, changes in the plant C:N are also related to the plant growth rate, and the plant C:N reflects the plant growth rate and plant nitrogen use efficiency (Han et al., 2014; Sun et al., 2016). Studies have confirmed that the late- species are characterized by slow growth (Li et al., 2015), indicating that these species use nitrogen more efficiently in nutrient-poor environments (Loess Plateau), leading to an increased plant C:N. Our results indicate that during PSF, plants undergo transformation to adapt to nitrogen assimilation and carbon fixation.

The plant C:P and N:P were higher in the second and third growth periods than in the first growth period (except for early- species in mid soil). Plant C:P and N:P are significantly affected by the plant’s ability to absorb and assimilate nutrients (Sistla and Schimel, 2012; Frazao et al., 2020). In this study, the decreased in soil phosphorus may have limited the absorption and utilization of phosphorus by plants (Supplementary Figures 3, 4) and thus increased the plant C:P and N:P. Meanwhile, RDA showed that the soil nutrient content of early- and mid- species was negatively correlated with plant C:P and N:P, which was supported by the overall decrease in SOC and TN content (Supplementary Figure 5) of early- and mid- species. The plant N:P reflects the plant nitrogen and phosphorus limitation (Braakhekke and Hooftman, 1999). In this study, the plant N:P was less than 14 (except for in early- species in early soil) in the first growth period and greater than 16 in the second and third growth periods, indicating that during the three growth periods, the plant transitions from a nitrogen-limited to a phosphorus-limited state in the PSF process.

### Soil C-N-P stoichiometry response to plant-soil feedback

We found that during the PSF process, soil C:N was in a balanced state and was not affected by the plant growth period. Stable changes in soil C:N are related to consistent changes in SOC and TN (Supplementary Figure 5; Cleveland and Liptzin, 2007). The soil C:P and N:P of early- and mid- species in early and late soil, and the soil C:P and N:P of late- species in early and mid-soil increased in the third growth period, possibly because of differences in the absorption and release of SOC, TN, and TP (Supplementary Table 6; Martin-Sanchez et al., 2020). Meanwhile, the soil C:P and N:P were overall negatively correlated with soil enzyme activities. Thus, during PSF, the decrease in soil enzyme activities (Supplementary Figure 6) may have limited the phosphorus circulation rate and increased the soil C:P and N:P. Soil C:P can reflect the availability of soil phosphorus (Michaels, 2003). Our results indicated that in the PSF process, the assimilation of soil microorganisms to soil phosphorus increased during plant growth period, thereby enhanced the soil’s ability to fix phosphorus. The soil N:P (0.65–2.48) of the three soils was lower than the national average of 5.2 (Tian et al., 2010), supporting that soil nitrogen is a limiting factor during the PSF process. However, we found that the nitrogen-limiting effect of the soil gradually decreased during plant growth period.

### Enzyme C-N-P stoichiometry and microbial nutrient limitation response to plant-soil feedback

Our study demonstrated that the enzyme C:N of early- species in early soil; enzyme C:N of mid- species in early, mid, and late soils; and enzyme C:N of late- species in early and mid-soils overall decreased in the third growth period. Cui et al. (2018) confirmed that the enzyme C:N changes mainly because of inconsistent changes in carbon- and nitrogen- acquiring enzyme activities. In this study, carbon-acquiring enzymes showed greater variation compared to nitrogen-acquiring enzymes from the second to third growth periods (Supplementary Table 7). Meanwhile, the lower SOC content of early- and mid- species in the third growth period may increase the microbial carbon limitation (Supplementary Figure 5; Camenzind et al., 2019). However, in late species, the SOC content increased in the third growth period, whereas the soil enzyme activity decreased, possibly because changes in SOC lag behind those of soil microorganisms and thus increase the sensitivity of the soil microbial response to soil nutrients during PSF, particularly in late- species (Bever et al., 1997; Zhang et al., 2021).

The enzyme C:P of early- species in early soil; enzyme C:P of mid- species in early, mid and late soil; and enzyme C:P of late- species in early and late soil were significantly reduced in the third growth period. This reduction occurred mainly because of the higher variation in carbon-acquiring enzymes and little or no variation in phosphorus-acquiring enzymes from the second to the third growth period (Supplementary Table 7). During the PSF process, the microbial carbon- limitation significantly decreased in the third growth period, because the SOC content and enzyme activity decreased in early- and mid- species soil in the third growth period, whereas the variation in SOC was lower than that in enzyme activity (Supplementary Table 6); In contrast, the SOC content of late- species soil significantly increased during the growth period, which effectively increased the input of carbon sources in the soil, thereby alleviating the soil microbial carbon limitation. In addition, our study indicated that microbial are also nitrogen-limited in the PSF process, and also a significant positive correlation between the vector angle and vector length was found in this study; in other words, in the PSF process, microbial are jointly limited by carbon and nitrogen. This is because in the process of plant growth, when soil microbes are limited by carbon, microbial metabolism gradually weakens, thereby slowing the absorption and utilization of nutrients (Xu et al., 2021b). Our research also found that the nitrogen limitations of all soils planted with the mid- and late- successional plant species were intensified in the third growth period, indicating that while the carbon limitation was alleviated by soil microorganisms, the nitrogen limitation of microorganisms was intensified.

### Plant-soil-enzyme stoichiometry cannot effectively explain the plant-soil feedback

During PSF, plant growth alters the soil chemical and biological characteristics, which can promote or inhibit plant growth (Gundale et al., 2019). Therefore, exploring the correlation among the plant-soil-enzyme C-N-P stoichiometry is important for understanding the PSF process. However, the plant-soil-enzyme stoichiometry showed no significant correlations. PSF is a complex process affected by the species type, climatic conditions, pH, and biological factors (Png et al., 2018; Crawford et al., 2019). Therefore, studies of PSF are needed to examine the correlation between plant-soil-enzyme stoichiometry and influencing factors under different environmental conditions.

Our results also indicated that early-, mid-, and late- species had negative, neutral, and positive PSF effects during the plant growth period. From the perspective of plant-soil-enzyme stoichiometry and nutrient limitation, the PSF effect of different succession species cannot be well explained. A possible reason is that the plant-soil-enzyme stoichiometry is more sensitive to environmental changes than to nutrients (Li et al., 2020); however, the stoichiometric changes, nutrient limitations, and their mechanisms in the ecosystem are not well-understood. Therefore, more comprehensive studies of changes in the plant-soil-enzyme stoichiometry and driving mechanisms of the PSF process are needed.

## Conclusion

In this 3-year PSF experiment, we detected a transformation–adaptation state in the assimilation of nitrogen and fixation of carbon by three successional plant species. The plant growth period mainly impacted the plant C:N in early- and late- species but had a lower impact on mid- species. Meanwhile, plant C:P and N:P overall increased across the plant growth period, and as the plant growth period progressed, the plants changed from a nitrogen-limited to a phosphorus-limited state. The plant growth period had less impact on soil C:N, and the microbial were limited by both carbon and nitrogen. The carbon limitation of microbial gradually decreased during the plant growth period. In addition, because of the complexity of the PSF process, the PSF effect during succession could not be effectively explained from the perspective of plant-soil-enzyme stoichiometry and microbial nutrient limitations. Our research improves the understanding of the nutrient absorption and limitation of plants, soil, and microorganisms in the PSF process.

## Data availability statement

The original contributions presented in this study are included in the article/Supplementary material, further inquiries can be directed to the corresponding author/s.

## Author contributions

HX, QQ, SX, and ZX designed the experiment. HX, QQ, and ZW performed the experiments and analyzed the data. HX wrote the manuscript. All authors made important contributions to the manuscript, and approved publication.
