# Supplementary material for: Plant-soil-enzyme C-N-P stoichiometry and microbial nutrient limitation responses to plant-soil feedbacks during community succession: A 3-year pot experiment in China
Source: Front Plant Sci. 2022 Sep 20;13:1009886. doi: 10.3389/fpls.2022.1009886 (PMC9531649; doi:10.3389/fpls.2022.1009886)
Supplement: Supplementary file 1 [file Data_Sheet_1.docx]

Table S1 Geographical of different succession stages species plots.

| Succession stage | Plots | Species | Latitude (E) | Longitude (N) | Elevation(m) | Years since abandonment | Growth habit |  |
| --- | --- | --- | --- | --- | --- | --- | --- | --- |
| Early- | P1 | *Setaria viridis* | 36°51′28″ | 109°19′37″ | 1254 | 1 | annual herb |  |
|  | P2 | *Setaria viridis* | 36°44′41″ | 109°16′04″ | 1264 | 1 | annual herb |  |
|  | P3 | *Setaria viridis* | 36°44′40″ | 109°15′36″ | 1259 | 2 | annual herb |  |
|  | P4 | *Setaria viridis* | 36°44′41″ | 109°16′02″ | 1262 | 2 | annual herb |  |
|  | P5 | *Setaria viridis* | 36°44′29″ | 109°15′22″ | 1276 | 2 | annual herb |  |
| Mid- | P6 | *Stipa bungeana* | 36°51′18″ | 109°19′19″ | 1300 | 13 | perennial herb |  |
|  | P7 | *Stipa bungeana* | 36°45′17″ | 109°19′08″ | 1221 | 15 | perennial herb |  |
|  | P8 | *Stipa bungeana* | 36°45′19″ | 109°15′35″ | 1194 | 15 | perennial herb |  |
|  | P9 | *Stipa bungeana* | 36°51′39″ | 109°18′46″ | 1036 | 18 | perennial herb |  |
|  | P10 | *Stipa bungeana* | 36°52′48″ | 109°19′04″ | 1303 | 20 | perennial herb |  |
| Late- | P11 | *Bothriochloa ischaemum* | 36°51′02″ | 109°18′52″ | 1269 | 30 | perennial herb |  |
|  | P12 | *Bothriochloa ischaemum* | 36°51′14″ | 109°19′16″ | 1293 | 33 | perennial herb |  |
|  | P13 | *Bothriochloa ischaemum* | 36°46′31″ | 109°16′39″ | 1290 | 34 | perennial herb |  |
|  | P14 | *Bothriochloa ischaemum* | 36°51′09″ | 109°19′05″ | 1298 | 34 | perennial herb |  |
|  | P15 | *Bothriochloa ischaemum* | 36°51′12″ | 109°19′08″ | 1290 | 35 | perennial herb |  |

Table S2 Mean (± SE) (n = 5) of soil chemical and biological properties of field soils that support early-, mid-, and late- species.

| Soil origin | Soil organic carbon  (g kg^-1^) | Total nitrogen  (g kg^-1^) | Total phosphorus  (g kg^-1^) | Available phosphorus  (mg kg^-1^) | Available nitrogen  (mg kg^-1^) | Microbial biomass carbon  (mg kg^-1^) |
| --- | --- | --- | --- | --- | --- | --- |
| Early- | 5.23±0.02a | 0.74±0.01a | 0.58±0.01a | 2.50±0.08a | 8.57±0.05a | 145.69±2.07a |
| Mid- | 4.48±0.01b | 0.51±0.02c | 0.56±0.00a | 1.90±0.03c | 6.29±0.06c | 93.38±1.90c |
| Late- | 5.18±0.01a | 0.64±0.01b | 0.46±0.01b | 2.20±0.03b | 8.14±0.04b | 115.39±0.95b |
|  | Microbial biomass nitrogen  (mg kg^-1^) | BG  (μmol h^-1^ g^-1^) | CBH  (μmol h^-1^ g^-1^) | NAG  (μmol h^-1^ g^-1^) | LAP  (μmol h^-1^ g^-1^) | AP  (μmol h^-1^ g^-1^) |
| Early- | 33.15±0.32a | 3.10±0.06a | 0.25±0.002a | 0.14±0.004a | 4.11±0.10a | 26.20±0.88a |
| Mid- | 29.54±0.45b | 2.68±0.01c | 0.21±0.010b | 0.09±0.002c | 3.54±0.04b | 20.92±0.85b |
| Late- | 28.45±0.18b | 2.91±0.01b | 0.23±0.004b | 0.11±0.001b | 3.68±0.14ab | 23.75±0.28ab |

Notes: BG, β-1, 4-glucosidase. CBH, β-D-cellobiosidase. NAG, β-1, 4-N-acetylglucosaminidase. LAP, L-leucine aminopeptidase. AP, acid phosphatase. Different letters indicate significant differences of the soil chemical and biological properties among the three growth period groups based on Duncan’s post hoc test (P < 0.05).

Table S3 Planting time and method.

| Plant growth period | Species | Planting time | Planting method | Harvest time |
| --- | --- | --- | --- | --- |
| First | *Setaria viridis* | 4-May-2018 | Planted in the first year: approximately 10 seeds of each species were sown | 4-Sep-2018 |
|  | *Stipa bungeana* | 4-May-2018 | Planted in the first year: approximately 10 seeds of each species were sown |  |
|  | *Bothriochloa ischaemum* | 4-May-2018 | Planted in the first year: approximately 10 seeds of each species were sown |  |
| Second | *Setaria viridis* | 4-May-2019 | Replanted | 4-Sep-2019 |
|  | *Stipa bungeana* | 4-May-2019 | Regrown from the previous year's root |  |
|  | *Bothriochloa ischaemum* | 4-May-2019 | Regrown from the previous year's root |  |
| Third | *Setaria viridis* | 4-May-2020 | Replanted | 4-Sep-2020 |
|  | *Stipa bungeana* | 4-May-2020 | Regrown from the previous year's root |  |
|  | *Bothriochloa ischaemum* | 4-May-2020 | Regrown from the previous year's root |  |

Notes: Because *Setaria viridis* is an annual plant, we replanted this species on May 4 of each year while retaining the root.

Table S4 F and p values of plant growth period to various parameters studied by a one-way ANOVA.

| Species | Parameter | Early species soil | | Mid species soil | | Late species soil | | Total | |
| --- | --- | --- | --- | --- | --- | --- | --- | --- | --- |
|  |  | *F* | *p* | *F* | *p* | *F* | *p* | *F* | *p* |
| Early- | Plant C:N | 6.58 | 0.012 | 0.96 | 0.410 | 0.46 | 0.642 | 2.44 | 0.129 |
|  | Plant C:P | 6.08 | 0.018 | 150.35 | <0.001 | 17.54 | <0.001 | 25.59 | <0.001 |
|  | Plant N:P | 1.47 | 0.268 | 62.55 | <0.001 | 19.54 | <0.001 | 14.88 | 0.001 |
|  | Soil C:N | 2.13 | 0.161 | 0.12 | 0.884 | 0.00 | 1.000 | 1.60 | 0.241 |
|  | Soil C:P | 9.64 | 0.003 | 6.27 | 0.014 | 43.46 | <0.001 | 220.40 | <0.001 |
|  | Soil N:P | 12.90 | 0.001 | 2.07 | 0.170 | 15.20 | 0.001 | 187.26 | <0.001 |
|  | Enzyme C:N | 6.22 | 0.014 | 1.55 | 0.252 | 3.20 | 0.077 | 1.93 | 0.188 |
|  | Enzyme C:P | 29.21 | <0.001 | 7.09 | 0.009 | 1.43 | 0.277 | 8.18 | 0.006 |
|  | Enzyme N:P | 50.99 | <0.001 | 5.39 | 0.021 | 1.73 | 0.219 | 12.79 | 0.001 |
|  | Vector length | 12.49 | 0.001 | 5.31 | 0.022 | 10.07 | 0.003 | 9.42 | 0.003 |
|  | Vector angle | 31.70 | <0.001 | 4.30 | 0.039 | 2.19 | 0.154 | 8.90 | 0.004 |
| Mid- | Plant C:N | 0.31 | 0.736 | 0.03 | 0.970 | 12.69 | 0.001 | 0.68 | 0.525 |
|  | Plant C:P | 64.54 | <0.001 | 49.77 | <0.001 | 26.29 | <0.001 | 167.88 | <0.001 |
|  | Plant N:P | 61.77 | <0.001 | 110.62 | <0.001 | 26.27 | <0.001 | 154.39 | <0.001 |
|  | Soil C:N | 0.98 | 0.403 | 11.37 | 0.002 | 0.21 | 0.816 | 0.43 | 0.659 |
|  | Soil C:P | 36.78 | <0.001 | 6.98 | 0.010 | 12.61 | 0.001 | 33.89 | <0.001 |
|  | Soil N:P | 19.45 | <0.001 | 3.97 | 0.047 | 8.58 | 0.005 | 72.58 | <0.001 |
|  | Enzyme C:N | 17.98 | <0.001 | 11.24 | 0.002 | 8.08 | 0.006 | 25.28 | <0.001 |
|  | Enzyme C:P | 5.83 | 0.017 | 9.46 | 0.003 | 20.40 | <0.001 | 11.42 | 0.002 |
|  | Enzyme N:P | 1.52 | 0.258 | 4.21 | 0.041 | 11.57 | 0.002 | 0.39 | 0.687 |
|  | Vector length | 33.64 | <0.001 | 16.78 | <0.001 | 23.54 | <0.001 | 102.49 | <0.001 |
|  | Vector angle | 4.66 | 0.029 | 1.17 | 0.344 | 25.16 | <0.001 | 39.41 | <0.001 |
| Late- | Plant C:N | 6.18 | 0.016 | 2.59 | 0.116 | 0.02 | 0.981 | 0.29 | 0.784 |
|  | Plant C:P | 52.73 | <0.001 | 39.30 | <0.001 | 26.33 | <0.001 | 86.92 | <0.001 |
|  | Plant N:P | 18.02 | <0.001 | 55.48 | <0.001 | 24.27 | <0.001 | 75.43 | <0.001 |
|  | Soil C:N | 8.96 | 0.004 | 1.43 | 0.278 | 0.49 | 0.625 | 4.79 | 0.030 |
|  | Soil C:P | 35.50 | <0.001 | 12.31 | 0.001 | 0.09 | 0.915 | 27.01 | <0.001 |
|  | Soil N:P | 16.16 | <0.001 | 17.38 | <0.001 | 0.28 | 0.761 | 9.46 | 0.003 |
|  | Enzyme C:N | 10.67 | 0.002 | 4.67 | 0.029 | 8.64 | 0.005 | 2.39 | 0.134 |
|  | Enzyme C:P | 17.20 | <0.001 | 5.22 | 0.021 | 70.05 | <0.001 | 9.45 | 0.003 |
|  | Enzyme N:P | 4.37 | 0.032 | 0.23 | 0.796 | 21.48 | <0.001 | 3.97 | 0.048 |
|  | Vector length | 30.66 | <0.001 | 8.97 | 0.004 | 542.74 | <0.001 | 62.32 | <0.001 |
|  | Vector angle | 4.35 | 0.032 | 4.96 | 0.027 | 13.44 | 0.001 | 24.05 | <0.001 |

Table S5 F and *p* values of axis studied by redundancy analysis (RDA).

|  | Early- species | | Mid- species | | Late- species | |
| --- | --- | --- | --- | --- | --- | --- |
|  | *F* | *p* | *F* | *p* | *F* | *p* |
| Axis1 | 34.17 | 0.004 | 30.72 | 0.005 | 58.00 | <0.001 |
| Axis 2 | 5.12 | 0.841 | 5.43 | 0.818 | 9.53 | 0.249 |
| Axis 3 | 1.44 | 1.00 | 1.09 | 1.00 | 3.37 | 0.933 |
| Axis 4 | 0.89 | 1.00 | 0.79 | 1.00 | 0.58 | 1.00 |

Table S6 Percentage changes in soil organic carbon, total nitrogen, and total phosphorus from the second to the third growth period (%).

| Species | Early soil | | | Mid soil | | | Late soil | | |
| --- | --- | --- | --- | --- | --- | --- | --- | --- | --- |
|  | Carbon | Nitrogen | Phosphorus | Carbon | Nitrogen | Phosphorus | Carbon | Nitrogen | Phosphorus |
| Early- | 16.5 | 3.7 | 44.1 | 23.4 | 34.4 | 51.9 | +7.2 | +6.5 | 9.4 |
| Mid- | 3.5 | 2.9 | 10.1 | 26.9 | 6.5 | 10.6 | +21.0 | +17.7 | 18.0 |
| Late- | 6.2 | 5.7 | 52.9 | 7.2 | 10.5 | 50.7 | +6.9 | +2.6 | +4.6 |

Notes: +, indicates increase.

Table S7 Percentage changes in carbon, nitrogen, and phosphorus acquisition enzymes from the second to the third growth period (%).

| Species | Early soil | | | Mid soil | | | Late soil | | |
| --- | --- | --- | --- | --- | --- | --- | --- | --- | --- |
|  | Carbon enzyme | Nitrogen enzyme | Phosphorus enzyme | Carbon enzyme | Nitrogen enzyme | Phosphorus enzyme | Carbon enzyme | Nitrogen enzyme | Phosphorus enzyme |
| Early- | 31.5 | 20.5 | +156.2 | 56.8 | 20.8 | 23.7 | 55.2 | 32.8 | 28.3 |
| Mid- | 13.6 | 0.4 | 23.0 | 49.4 | 22.9 | 23.9 | 56.6 | 46.6 | 53.2 |
| Late- | 23.3 | 11.1 | 25.4 | 52.1 | 26.8 | 24.5 | 46.3 | 46.0 | +7.3 |

Notes: +, indicates increase.


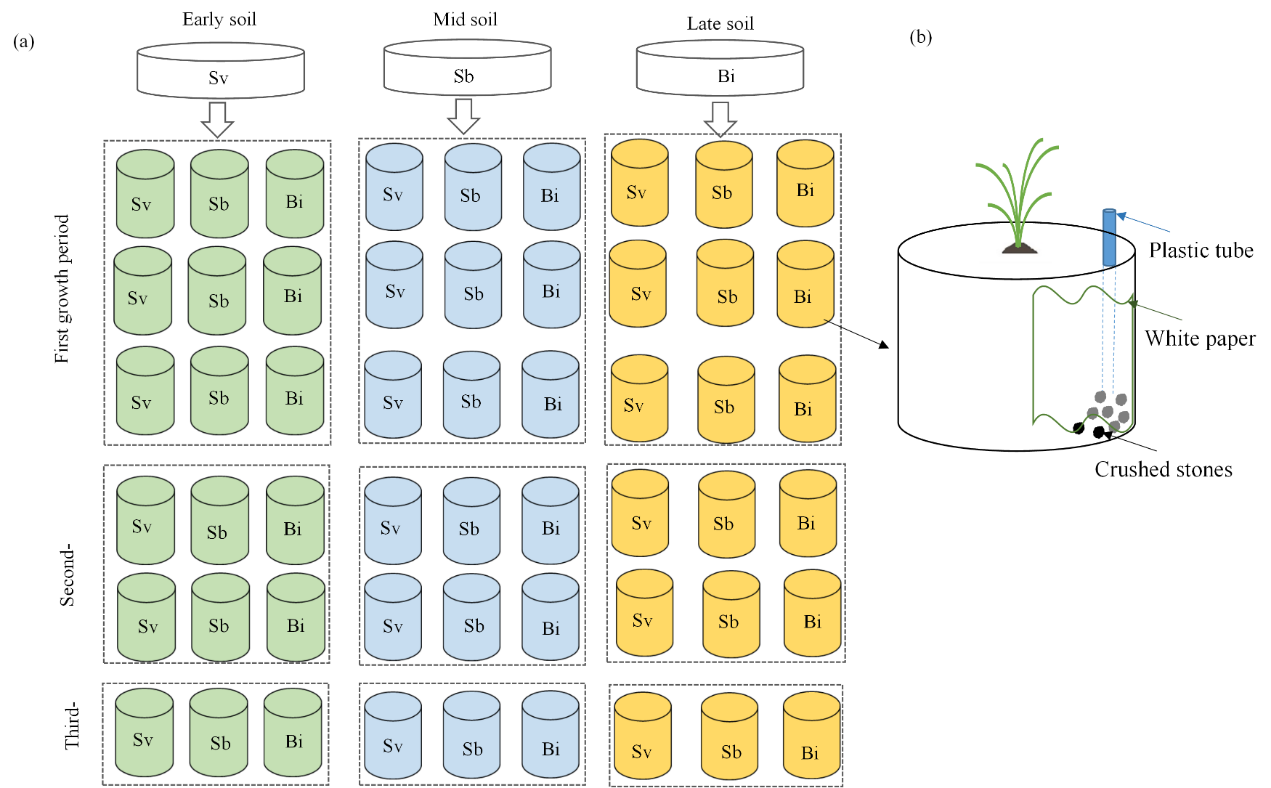


Figure S1 Experimental design and planting scheme. Notes: field soil of early, mid, and late succession were placed in pots for *Setaria* *viridis* (Sv), *Stipa* *bungeana* (Sb), and *Bothriochloa* *ischaemum* (Bi) for cultivation. All plants were grown for four months.


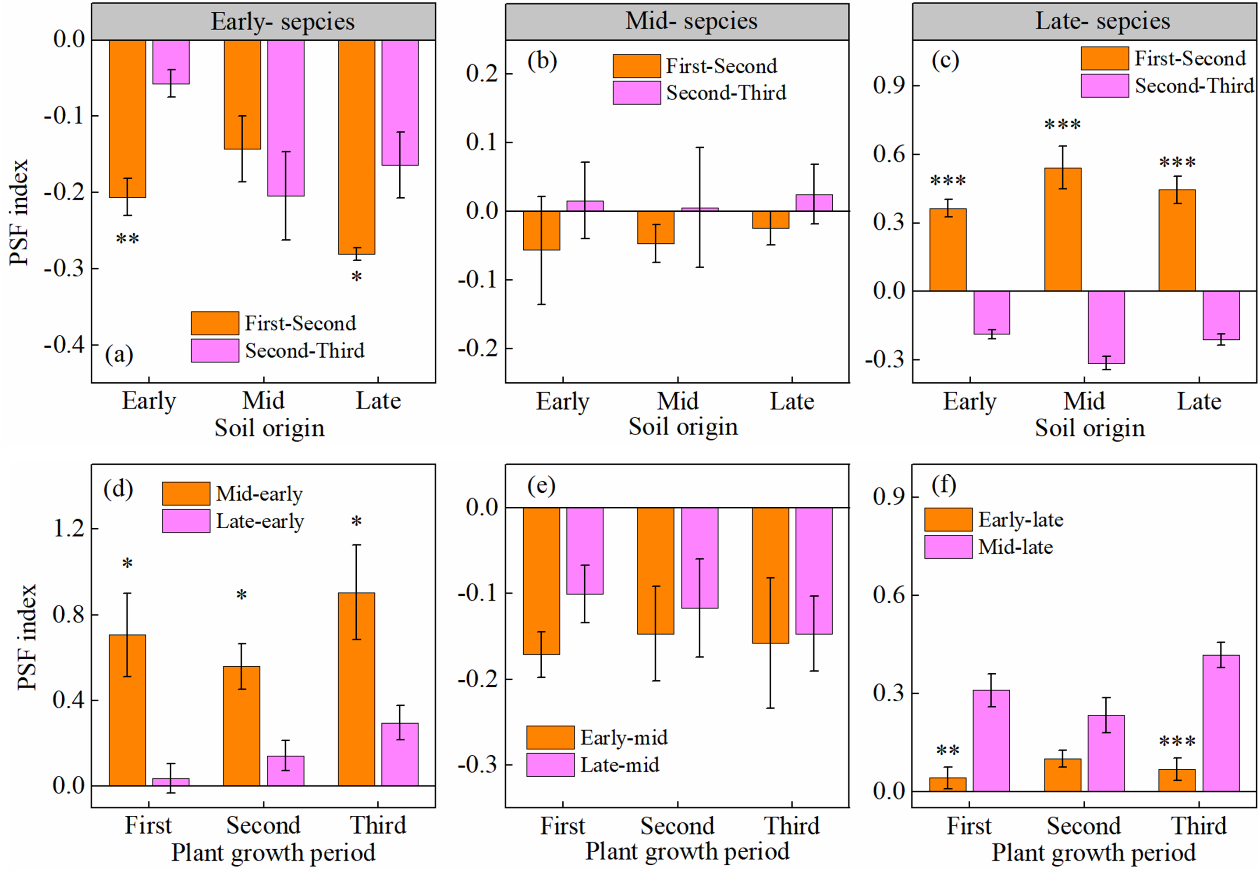


Figure S2 Mean (±SE) (n=5) plant-soil feedback (PSF) index of early- (a), mid- (b), and late- species (c). Note: First-Second is the PSF index from the first to second growth period. Second-Third is the PSF index from the second to third growth period. Asterisks indicate significant differences in PSF index between different treatments based on t-Test. ***: *p*<0.001. **: *p*<0.01. *: *p*<0.05.





Figure S3 The relationships between vector length and vector angle. Note: Data points are the means of vector length and vector angle of each treatment.





Figure S4 Mean (±SE) (n=5) plant carbon, nitrogen, and phosphorus content of early- (a-c), mid- (d-f), and late- species (g-i) over the three growth periods. Note: Different lower letters indicate significant differences in mean plant carbon, nitrogen, and phosphorus content among the three growth periods based on Duncan’s post hoc test (*P* < 0.05).





Figure S5 Mean (±SE) (n=5) soil organic carbon (SOC), total nitrogen (TN), and total phosphorus (TP) of early- (a-c), mid- (d-f), and late- species (g-i) over the three growth periods. Note: Different lower letters indicate significant differences in mean SOC, TN, and TP among the three growth periods based on Duncan’s post hoc test (*P* < 0.05).





Figure S6 Mean (±SE) (n=5) soil enzyme activity of early- (a-e), mid- (f-j), and late- species (k-o) over the three growth periods. Note: BG, β-1,4-glucosidase. NAG, β-1,4-N-Acetylglucosaminidase. CBH, β-D-Cellobiosidase. MAP, Acid phosphatase. LAP, L-Leucine aminopeptidase. Different lower letters indicate significant differences in mean soil enzyme activity among the three growth periods based on Duncan’s post hoc test (*P* < 0.05).
